# The interrelationship of mycophagous small mammals and ectomycorrhizal fungi in primeval, disturbed and managed Central European mountainous forests

**DOI:** 10.1007/s00442-012-2303-2

**Published:** 2012-04-01

**Authors:** Susanne Schickmann, Alexander Urban, Katharina Kräutler, Ursula Nopp-Mayr, Klaus Hackländer

**Affiliations:** 1 Department of Integrative Biology and Biodiversity Research, Institute of Wildlife Biology and Game Management, University of Natural Resources and Life Sciences, Gregor-Mendel-Strasse 33, 1180 Vienna, Austria; 2 Department of Systematic and Evolutionary Botany, Faculty of Life Sciences, University of Vienna, Rennweg 14, 1030 Vienna, Austria; 3 Department of Forest and Soil Sciences, Institute of Forest Entomology, Forest Pathology and Forest Protection, University of Natural Resources and Life Sciences, Hasenauerstraße 38, 1180 Vienna, Austria

**Keywords:** Rodents, Shrews, Truffles, Mutualism, Nestedness

## Abstract

Small forest dwelling mammals are considered to be major consumers and vectors of hypogeous ectomycorrhizal (ECM) fungi, which have lost the ability of active spore discharge. Fungal spore dispersal by mycophagy is deemed an important process involved in forest regeneration, resilience and vitality, primarily based on evidence from Australia and the Pacific Northwestern USA, but is poorly known for Central European mountainous forests thus far. Small mammal mycophagy was investigated by live trapping and microscopical analysis of faecal samples. All small mammal species recorded (*Myodes glareolus*, *Microtus agrestis*, *Pitymys subterraneus*, *Apodemus* spp., *Glis glis*, *Sorex* spp.) had ingested spores of ECM fungi, albeit in varying amounts. *My. glareolus* was found to be the most important vector of ECM fungal spores, both in quantity and diversity. Species of the genus *Sorex* seem to play a hitherto underestimated role as dispersers of fungal spores. *Glis glis* is likely to be an important vector owing to its large home range. Hypogeous ECM basidiomycetes accounted for most spores found in the faecal samples. The frequency of various genera of hypogeous ECM ascomycetes and ECM epigeous fungi was much lower. Comparison with null models indicated a non-random structure of the mycophagy network similar to other mutualistic bipartite networks. Mycophagy can be considered (1) to contribute to nutrition of small forest mammals, (2) to play a pivotal role for forest regeneration and functioning by providing mycorrhizal inoculum to tree seedlings and (3) to be vital for reproduction and diversity of the still poorly known hypogeous fungi.

**Electronic supplementary material:**

The online version of this article (doi:10.1007/s00442-012-2303-2) contains supplementary material, which is available to authorized users.

## Introduction

Mycophagy, the use of fungi as a food source, is recognised as a feeding habit of many animal species in different ecosystems as well as a major way of spore dispersal for hypogeous fungi, which almost exclusively form ectomycorrhizae (ECM) (Fogel and Trappe 1978; Johnson 1996; Vernes and Dunn 2009). Hypogeous fungi lack mechanisms of spore discharge to the air (Fogel and Trappe 1978) and reconstructions of fungal phylogenies suggest that the hypogeous fruiting habit evolved repeatedly from epigeously fruiting genera in ascomycetes and basidiomycetes (Johnson 1996; Trappe and Claridge 2005). This implies that endozoochory is a successful way of spore dispersal. Higher independence from weather conditions, the pelleting of spores in nutrient reserves attractive for fine roots and the visiting of favourable habitats by fungivorous mammals are the major advantages of this tripartite mutualism involving mycorrhizal plants, mycorrhizal fungi and fungivorous animals (Johnson 1996). In turn, mycophagists can indirectly influence vegetation succession by dispersing propagules of mycorrhizal fungi, thereby promoting the spread and regeneration of obligately mycorrhizal plant species (Bruns 1995; Terwilliger and Pastor 1999; Wiemken and Boller 2006). Maser et al. (2008) hypothesize that without animal dispersal of spores of hypogeous fungi, growth, regeneration and adaptation of the mycorrhizal fungi–tree network would be greatly impaired if not impossible.

Many animal species in different ecosystems have been shown to be mycophagous (Ure and Maser 1982; Claridge and Lindenmayer 1998; Tuno 1998; Paugy et al. 2004; Jones et al. 2007). Maser et al. (2008) classify mycophagists as obligate (e.g. *Myodes californicus*, *Potorous longipes*); preferential (e.g. *Glaucomys sabrinus*, *Bettongia penicillata*); opportunistic (e.g. *Peromyscus* sp*.*, *Oreamnos americanus*, *Alces alces*, *Wallabia bicolor*) or accidental mycophagists (e.g. predators of mycophagists, *Dasyurus* sp., *Strigiformes*) according to their degree of mycophagy.

Even though there are some data available from other biogeographic regions (e.g. Reddel et al. 1997; Mangan and Adler 2002), the majority of studies have been undertaken in the Pacific Northwestern USA and in Australia. For these two regions research has proven the pivotal role of mycophagy for both animal conservation and reproduction of a diverse range of hypogeous fungi (Cázares and Trappe 1994; Frank et al. 2006; Vernes and Dunn 2009), and has already led to management implications (Carey et al. 2002; Dell 2002; Wiensczyk et al. 2002). But, are the results achieved and the conclusions drawn also applicable to Central European mountainous forests with their specific plant, animal and fungal communities?

A few studies provide first insights. Drożdż (1966) observed that *My. glareolus* turns to mycophagy when beech seeds are sparse. Blaschke and Bäumler (1989) investigated mycophagy and spore dispersal of some small mammal species in Bavarian forests but did not analyse in detail the fungal species consumed. Grönwall and Pehrson (1984) and Bertolino et al. (2004) studied mycophagy of the red squirrel, and Wiemken and Boller (2006) investigated the role of ungulates as mycophagists. One very recent study from Lithuania (Kataržytė and Kutorga 2011) evaluated the degree of mycophagy of various small mammals in different forest types. They found that *Myodes glareolus* exhibited the highest numbers and diversity of fungal spores in faecal samples and that faeces of shrews contained more fungal spores than previously guessed.

Among the open questions related to mycophagy, the influence of natural disturbances and forest management practices on the community structures of both small mammals and ECM fungi, and on the degree of mycophagy (Carey et al. 2002; Jacobs and Luoma 2008) is still unknown in Central European mountainous forests. In primeval or sustainably managed forests, ECM occurrence is unlikely to limit tree growth or mycophagy owing to the typically high levels of mycorrhizal colonisation and ECM fungal diversity (Luoma et al. 2004). Conditions approximating primary succession (large-scale windthrows, avalanches, fires, floods or large-scale forest replacement by human activities) on the contrary might lead to the disruption of the mycorrhizal network (Perry et al. 1987). In such conditions, the recolonisation by trees might be limited by the availability of ECM fungi and aided by supply of germinable ECM spores through defecation (Cázares and Trappe 1994; Terwilliger and Pastor 1999; Wiemken and Boller 2006). To achieve a more complete understanding of regeneration and colonisation processes in forest ecosystems and adjacent areas, the vector function of animals in dispersal of ECM spores needs to be evaluated.

The specific structure of mycophagist–fungus interaction networks seems to be an open question, too. The relationship between mycophagists and fungi might be highly nested in ways that are known from plant–pollinator or plant–frugivore food webs (Bascompte et al. 2003), but could also be random, dependent only on fungal availability. Nested arrangement of food webs means that there is a core of species in both trophic levels interacting with a larger number of members of the other trophic level along with a number of more peripheral species interacting with a subset only. The specific structure of mutualistic networks influences species dispersal, persistence and coexistence (Bascompte et al. 2003).

To answer some of the pending questions regarding the mycophagist–fungi network we focus on four hypotheses regarding small mammal mycophagy and the fungal species consumed:

1. Central European small mammals inhabiting forested ecosystems show differences regarding degree of mycophagy and fungal species consumed.
2. Degree of mycophagy and fungal species consumed show seasonal and yearly variation.
3. Forest area (microhabitat) has an influence on degree of mycophagy and fungal species consumed.
4. The mycophagist–fungi interrelationship is non-random.

## Materials and methods

### Survey area and period

We collected small mammal faecal material in the Dürrenstein Wilderness Area, Austria (47° 48′ to 47° 45′ N, 15° 01′ to 15° 07′ E, 2,300 ha) and in the Rosalia Demonstration Forest, Austria (47° 42′ 0″ N, 16° 17′ 52″ E, 930 ha). Within the Dürrenstein Wilderness Area six survey plots (each about 0.8 ha) were studied, two each situated in Austria’s largest primeval forest (PF), in an adjacent managed forest (MF1) and in a disturbed area (DA; windthrown in 1990). A further two survey plots were studied in managed forest (MF2) in the Rosalia Demonstration Forest.

The Dürrenstein Wilderness Area is located in the eastern part of the Northern Limestone Alps. The climate is suboceanic–subcontinental with long winters and short, cool summers. Annual precipitation reaches about 2,000 mm with peaks during summer and winter months. Deep and wet snow cover is lasting, shortening the growing season (Splechtna et al. 2005).

The two PF plots were located in the primeval forest found in a watershed on the southern slopes of the Dürrenstein mountain (900–1,200 m a.s.l.). The forest vegetation is classified as Asperulo-Abieti-Fagetum and Adenostylo glabrae-Abieti-Fagetum (Splechtna et al. 2005). European beech (*Fagus sylvatica*) is the dominating tree species in the primeval forest, but the co-dominant European silver fir (*Abies alba*) and Norway spruce (*Picea abies*) grow 10–15 m taller, thus forming a two-layered canopy (Zukrigl et al. 1963). The amount of snags and downed coarse woody debris was estimated to be 82.6 and 134.2 m^3^ ha^−1^, respectively (Gratzer G., personal communication).

The two managed forest plots (MF1) were situated in spruce-dominated spruce-fir-beech forest. The distance to parts of the primeval forest was small, so climatic and geological parameters were the same, as was the potential natural vegetation. Contrary to the PF the mature trees were of rather uniform size and spacing. As a result of harvest activities the canopy was recently thinned, and we found higher cover of ground vegetation (tree regeneration, *Vaccinium myrtillus*, graminoids, ferns and mosses), abundant small woody debris and a limited amount of coarse woody debris.

The two disturbed area plots (DA) were situated on the southern slope of the Dürrenstein mountain. The general climatic and geological features as well as the potential natural vegetation were the same as for the primeval and managed forest plots, but microclimatic conditions differed because of the loss of tree canopy and the southern exposure of the site. We observed a locally dense cover of ground vegetation (graminoid, herb and perennial shrub species) and patches of dense tree regrowth dominated by beech or, more rarely, by spruce, with interspersed maple (*Acer pseudoplatanus*) or rowan (*Sorbus aucuparia*) in a tessellate pattern. Also, we encountered extraordinarily high amounts of coarse woody debris in different stages of decay as a result of the windthrow.

The other two investigation plots (MF2) were situated in the Rosalia Demonstration Forest in the Rosalia Mountains, at the northeastern margin of the Alps. Reaching from 350 to 750 m a.s.l., the area is characterized by moderate winters, warm summers and an average annual precipitation of 800 mm. There are beech-dominated forest stands as well as spruce-fir-beech forests at higher elevations and northern slopes (Marschall and Sagl 1986). The investigated forest plots (MF2) were located between 600 and 700 m a.s.l. and characterised by spruce-fir-beech forest with an understory mainly consisting of beech regeneration and various graminoids, ferns and perennial shrubs (*Rubus* spp., *Atropa belladonna*). There were no signs of recent logging; small woody debris and a low amount of coarse woody debris were present throughout the plots.

We live trapped small mammals in accordance with international standards (Gannon and Sikes 2007) on all eight plots in summer and autumn 2006 and 2007, resulting in four trapping sessions. We sampled every plot once for three consecutive nights per trapping session.

### Trapping and sampling procedure

We arrayed traps in a five by five grid, spaced 15 × 15 m and placed two traps at every station. Traps were equipped with peanut butter cookies, apple slices, rodent chow, mealworms, and hay as bait, food, and thermal insulation. After each capture we thoroughly cleaned the traps, refilled and reset them in the same spot. We identified animals on the basis of morphological traits (Corbet et al. 1982) and marked them for recapture recognition. Permission and method approval were obtained from the administrations concerned prior to trapping.

From each newly captured animal we took faecal pellets from the trap. We sampled each animal once only and stored samples in Eppendorf reaction tubes (1.5 ml) filled with 1 ml silica gel beads as desiccant.

### Microscopical analysis

We separated dry pellets from debris and silica gel beads, then suspended and macerated each sample in 600 μl distilled water, and kept samples frozen at −20 °C for further use.

We transferred two drops of the thoroughly homogenised sample solution with broad-bladed forceps onto a microscopic slide, setting the drops apart from one another. We then added one drop of Melzer’s reagent (Morton 1989) to one subsample to stain the polysaccharide components of the fungal spores. We mounted the other subsample with Hoyer’s mounting medium (Cunningham 1972). After separate homogenisation with a preparation needle, we covered subsamples with a coverslip and sealed them with nail polish to prevent evaporation.

We used a Reichert Polyvar light microscope with 100–1,000-fold magnification and an affixed Nikon D70 digital camera. We analysed all faecal samples separately, selecting 25 random fields of view (fov) with 400-fold magnification in each of the two subsamples per slide. We counted all spores visible in the fov, allocating them to numbered spore types. We produced a detailed description of each spore type at 1,000-fold magnification according to Castellano et al. (1989). The identification of fungal spores was based on Montecchi and Sarasini (2000) and Castellano et al. (1989), and compared to DNA-based identification (Urban et al. in preparation).

### Calculation and statistics

Calculations were based on spore numbers and frequencies. As correction for unequal spore sizes we used volume units (based on average spore dimensions and a spheroid model of spore shape). But as this did not alter most of the results, we returned to the direct and more transparent measure of spore numbers and gave information on spore volume units where informative.

Fluctuations in small mammal capture frequencies resulted in highly varying sample sizes regarding small mammal species, trapping sessions and forest areas. We excluded the two rarest small mammal species (*N* = 2) and the rarest fungal spore types observed (spore count less than 50, never more than two spores per fov) from all calculations. We used the Kruskal–Wallis test followed by post hoc Mann–Whitney *U* tests for pairwise comparison and negative binomial regression to account for overdispersion, i.e. variance-to-mean ratio (VMR) = σ^2^μ^−1^ ≫ 1, non-normality and heterogeneity of variance in our data and analysed the four main and the four less frequent small mammal species separately to reduce the effects of variation in sample size. Variation in spore numbers of individual spore types was high and it was not possible to obtain a normally distributed dataset of individual spore types by transformation.

We used the number of spore types as measure of fungal species richness in small mammal food. By transformation (square root) we achieved a normal data distribution and therefore applied parametric tests (MANOVA, Tukey multiple comparison of means) to evaluate differences between small mammal species, capture session and forest type regarding species richness.

To compare the similarity of the distribution of fungal taxa found in samples from *My. glareolus* from all four forest areas we used a hierarchical cluster analysis (dissimilarity calculation with Bray–Curtis index and hierarchical clustering with nearest neighbour method using R package “vegan” (Oksanen et al. 2011).

For all statistical analyses, we used the open statistical package R (R Development Core Team 2011).

To calculate the degree of nestedness of the investigated mutualistic network we used the “nestedness” implementation in the R package “vegan” (Oksanen et al. 2011), which is a direct port of the binmatnest program (Rodríguez-Gironés and Santamaría 2006). In analogy to thermodynamics, the degree of nestedness as opposed to the entropy of a system is calculated as nestedness temperature. The lower the nestedness temperature, the more nested is the system. Comparison of the nestedness temperature calculated from empirical data and the temperature of three different randomly arranged null models is then used for estimation of the degree of nestedness of a given network. For visual representation of the interrelationships and nested arrangement, we generated a heat map, where the interactions between small mammal species and ECM fungi are represented as the product of the percentage of positive samples and the median spore number of positive samples. This value and the corresponding shade of grey provide an estimation of feeding intensity.

## Results

### Small mammal species and ECM fungal spore types

**Fig. 1:**
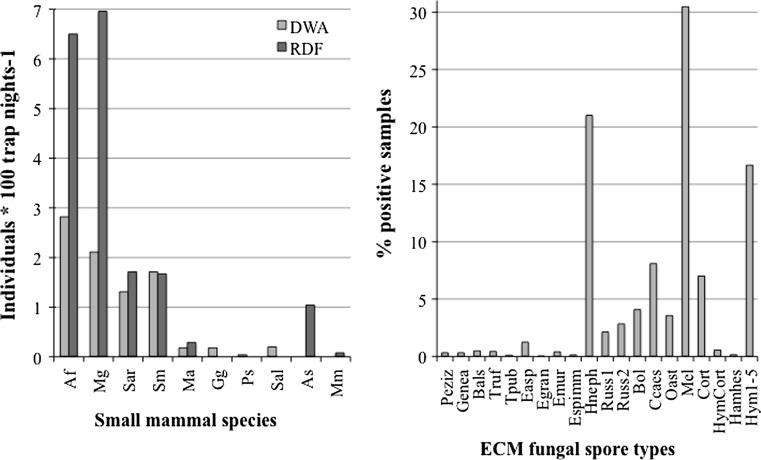
Small mammal community present in the investigated forest plots (*left*, *DWA* Dürrenstein Wilderness Area, *RDF* Rosalia Demonstration Forest) and ECM fungi present in small mammal faecal samples (*right*),* Af* *Apodemus flavicollis*,* Mg* *Myodes glareolus*,* Sar* *Sorex araneus*, *Sm S. minutus*, *Ma Microtus agrestis*,* Gg* *Glis glis*, *Ps Pitymys subterraneus*, *Sal S. alpinus*, *As A. sylvaticus*, *Mm Micromys minutus*; abbreviations of fungal taxa are defined in Table 1

During the live trapping sessions in the six plots representing three forest types of the Dürrenstein Wilderness Area we captured a total of 400 individuals of eight small mammal species. We achieved a trapping success (captures per 100 trap nights without recaptures) of 2.82 *Apodemus flavicollis*, 2.11 *Myodes glareolus*, 1.31 *Sorex araneus*, 1.71 *S. minutus*, 0.18 *Glis glis*, 0.18 *Microtus agrestis*, 0.20 *S. alpinus* and 0.04 *Pitymys subterraneus* (Fig. 1). The capture results show a typical Central European mountain forest small mammal community with four species comprising about 90 % of the captures and another four species captured in substantially lower numbers. The main four species are (1) *A. flavicollis*, a forest dwelling murid species with a broad ecological niche; (2) *My. glareolus*, an arvicolid species found in a wide range of forested habitats but favouring moist forests with dense understory and high amounts of coarse woody debris (Corbet et al. 1982); (3) *S. araneus* and (4) *S. minutus*, two frequent Eurasian forest soricids with a broad ecological niche (Mitchell-Jones et al. 1999). Less frequently occurring in the Dürrenstein Wilderness Area are *S. alpinus*, *G. glis*, and two other arvicolid species (*Mi. agrestis* and *P. subterraneus*) captured on the MF1 and DA plots.

In the Rosalia Demonstration Forest we captured 453 individuals of seven species (Fig. 1). Trapping success (100 trappings per night) was 6.50 *A. flavicollis*, 6.96 *My. glareolus*, 1.71 *S. araneus*, 1.67 *S. minutus*, 1.04 *A. sylvaticus*, 0.29 *Mi. agrestis* and 0.08 *Micromys minutus*.

The small mammal communities consist of the same four main species in both investigation areas, but differ in the accompanying species.

We analysed a total of 400 faecal samples (Fig. 1) from the three Dürrenstein Wilderness Area forest areas and 122 samples from the MF2 plots (selection based on sample number per species and session).

The sampling design allowed us to compare different forest areas as well as different sessions on a species-specific level for common small mammal species. To study the feeding ecology of less frequently captured small mammal species, prolonged sampling sessions or inclusion of recaptured animals in the sampling process would be necessary to obtain sufficient sample sizes.

During microscopic analysis of the faecal samples we identified 73 distinct spore types. After exclusion of 20 spore types (too rare), we confirmed 20 of the remaining 53 fungal spore types to represent distinct taxa of ECM fungi. Spores from the remaining 33 distinct spore types could not be assigned to any ectomycorrhizal fungal genus and amounted to 12 % of the total number of counted spores. They were excluded from further calculations.

The 20 ECM fungal spore types were present in varying amounts in the faecal samples (Fig. 1) and belonged to different phylogenetic groups (Table 1). All recorded ECM fungal taxa produce fleshy fruit bodies and thus constitute a potential food source for small mammals. We found 14 taxa of hypogeous fungi, two epigeous taxa and four which could not be assigned to either type of fruiting with certainty.

**Table 1:** Systematic classification and ecology of ECM fungal groups determined

	Order	Family	Type	Abbreviation	Fruiting habit	Ecology^a^	Found as sporocarp
*Asco* *mycota*	*Pezizales*	*Pyronema* *taceae*?	*Pezizales* sp*.*	Peziz	Epigeous?		
		*Pyronema* *taceae*	*Genea* sp*.*	Genea	Hypogeous		
		*Helvellaceae*	*Balsamia* sp*.*	Bals	Hypogeous		
		*Tuberaceae*	*Tuber rufum agg.*	Truf	Hypogeous	“Widespread in all environments, associated with both coniferous and deciduous trees, from spring to late autumn”	
			*Tuber* aff*. puberulum*	Tpub	Hypogeous		
	*Eurotiales*	*Elaphomy* *cetaceae*	*Elaphomyces* cf*. asperulus*	Easp	Hypogeous	“Not very common, preferably in coniferous or mixed woods, under *P. abies* or *A. alba*, more rarely in deciduous woods, growing all the year”	
			*Elaphomyces granulatus*	Egran	Hypogeous	“Common in coniferous woods, in autumn–winter, generally rather deep in the soil, under the needle sheet, up to 1,200 m altitude”	X
			*Elaphomyces* cf*. muricatus*	Emur	Hypogeous	“Rather common, in deciduous or coniferous woods, generally rather deep in the humus, mainly in summer and autumn, up to 1,200 m altitude”	
			*Elaphomyces* sp*. imm.*	Espimm	Hypogeous		
*Basidio* *mycota*	*Hysteran* *giales*	*Hysterangia* *ceae*	*Hysterangium nephriticum*	Hneph	Hypogeous	“Generally under deciduous trees, especially in woods with *F. sylvatica*, autumn”	X
	*Russulales*	*Russula* *ceae*	*Russulaceae 1*	Russ1	?		
			*Russulaceae 2*	Russ2	?		
	*Boletales*	*Boletaceae*	*Chamonixia caespitosa*	Ccaes	Hypogeous	“Quite rare species, found from July to October in mountain coniferous forests (*Picea*, *Abies*)”	
		*Melano* *gastraceae*	*Octaviania asterosperma*	Oast	Hypogeous	“Very frequent species, sometimes nearly semi-epigeous, in deciduous or mixed woods, at various altitudes and climates”	X
			*Boletaceae* sp*.* (*B. edulis*?)	Bol	Epigeous?		
			*Melanogaster broomeianus*	Mel	Hypogeous	“Very common species in all kind of fresh and shadowy woods, from the plain to the mountains, from spring to late autumn, mainly semi-epigeous”	
	*Agaricales*	*Cortinaria* *ceae/Hymeno* *gastraceae*	*Cortinarius* sp*.*	Cort	Epigeous		
			*Hymenogaster* sp*./Cortinarius* sp*.*	HymCort	Epigeous?		
			*Hymenogaster* cf*. hessei*	Hymhes	Hypogeous		
			*Hymenogaster* sp*.*1-5	Hym1-5	Hypogeous		

^a^Information about ecology is based on Montecchi and Sarasini (2000)

Ten of the 20 taxa comprised about 97 % of all observed ECM spores (Table 1 ESM), and among these ten three types representing hypogeous basidiomycetes (*Melanogaster broomeianus*, *Hysterangium nephriticum* and *Hymenogaster* sp.1–5) dominated with more than 15 % of counted spores each. The ratio of spore abundance of ascomycete genera (*Balsamia*, *Genea*, *Elaphomyces*, *Tuber*) basidiomycetes was 1:44, whereas the abundance ratio of hypogeous taxa and epigeous taxa was 15:1 (unclassified spore types excluded). The ratio of spore volume units for ascomycetes versus basidiomycetes was 1:2 and for hypogeous versus epigeous taxa it was 37:1.

### Degree of mycophagy of small mammals

**Fig. 2:**
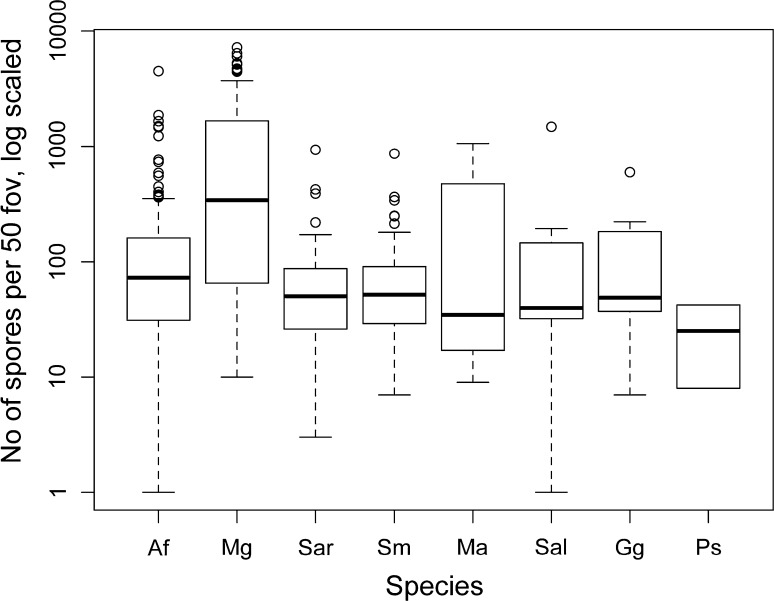
Boxplot of total ECM spore numbers (log scaled, zeros omitted) for small mammal species from DWA (small mammal species abbreviations as in Fig. 1), whisker length = 1.5 SD, outliers depicted as *open circles*

All investigated small mammal species had ingested ECM fungal spores (Table 2; Fig. 2), but we found considerable variation in median spore numbers between the four main small mammal species (Kruskal–Wallis chi-squared = 107.3, df = 3, *p* < 0.001). Post hoc pairwise comparison showed *My. glareolus* to defecate significantly higher numbers of ECM spores than the other three main species. *A. flavicollis* samples contained significantly more ECM fungal spores than samples from *S. araneus* and *S. minutus* (Table 2). Comparison of the samples from the four rarer small mammal species showed insignificant differences (Kruskal–Wallis chi-squared = 2.94, df = 3, *p* = 0.401; Table 2; Fig. 2).

**Table 2:** Number of samples (*N*), median number of ECM spores observed per 50 fov, and pairwise comparison of observed ECM spore numbers with Kruskal–Wallis post hoc procedure for eight small mammal species

Species	*N*	Total median no. of ECM spores/50 fov	Pairwise comparison with	*W*	*P* value
*My. glareolus*	167	79	*A. flavicollis*	18,789.5	**<0.001**
			*S. araneus*	8,930.5	**<0.001**
			*S. minutus*	12,319.0	**<0.001**
*A. flavicollis*	153	7	*My. glareolus*	7,763.5	**<0.001**
			*S. araneus*	6,516.0	**0.004**
			*S. minutus*	9,236.0	**<0.001**
*S. araneus*	66	4	*A. flavicollis*	3,978.0	**0.004**
			*My. glareolus*	2,091.5	**<0.001**
			*S. minutus*	3,171.5	0.395
*S. minutus*	89	4	*A. flavicollis*	4,915.0	**<0.001**
			*My. glareolus*	2,544.0	**<0.001**
			*S. araneus*	2,702.5	0.395
*A. sylvaticus*	8	4.5	*G. glis*	40.5	0.084
			*Mi. agrestis*	91.5	0.752
			*S. alpinus*	91.5	0.962
*G. glis*	8	11	*A. sylvaticus*	103.5	0.084
			*Mi. agrestis*	57.5	0.281
			*S. alpinus*	55.0	0.196
*Mi. agrestis*	11	8	*A. sylvaticus*	106.5	0.751
			*G. glis*	30.5	0.281
			*S. alpinus*	51.5	0.813
*S. alpinus*	11	5	*A. sylvaticus*	88.5	0.962
			*G. glis*	25.0	0.196
			*Mi. agrestis*	58.5	0.813

Significant differences indicated in bold

### Effect of session, year and forest area on total spore numbers for single small mammals species

Negative binomial regression indicated differing patterns of factor influence for each of the four main small mammal species. The regression model (total ECM fungal spore no. ~ plot + session + year) demonstrated a significant influence of “Plot” (forest type) on the degree of mycophagy of the two *Sorex* species: for *S. araneus* both “Plot” factor levels MF1 and PF and for *S. minutus* factor level MF1 had a significantly positive effect. Observed effects of “Plot” on *A. flavicollis* and *My. glareolus* were not significant. Factor influence of both “Session” and “Year” was significant for both rodent species, but not for *Sorex* spp. (Table 3). As a result of the small sample size of the four rare species and their confinement to single forest areas no statistically reliable comparison was possible.

**Table 3:** Coefficients of negative binomial regression model for total numbers of ECM spores for the four main small mammal species

Factor	*A. flavicollis*	*My. glareolus*	*S. araneus*	*S. minutus*
Intercept	−4.036***	−3.313***	0.518	1.876′
Plot				
DA	0	0	0	0
MF2	−1.061	−1.036	−0.225	0.195
MF1	0.644	0.973	4.576***	2.418*
PF	1.054	0.507	4.226***	0.854
Session				
Autumn	0	0	0	0
Summer	−4.516***	−0.347	−0.718	0.130
Year				
2006	0	0	0	0
2007	4.044***	3.324***	−0.516	−1.874′

Parameters that were significant at *P* = 0.05 are in bold

′* P* = 0.1; * P = 0.05; ** P = 0.01; *** P = 0.001

**Fig. 3:**
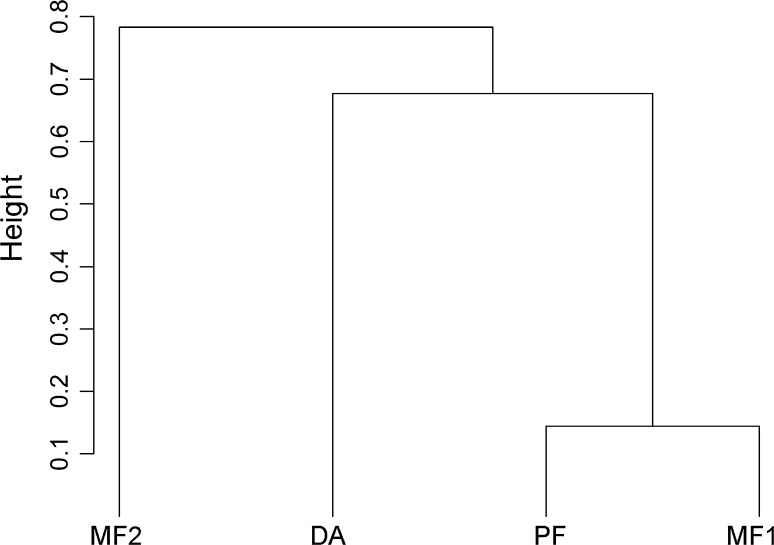
Hierarchical cluster analysis based on ECM fungal spores in *My. glareolus* samples from all four forest areas, clustering method—nearest neighbour, height—amount of dissimilarity, maximum = 1, default rooting

Fungal spore composition in samples from *My. glareolus* indicate site-specific ECM communities as illustrated by a hierarchical cluster analysis with samples from all four forest areas (Fig. 3). The dendrogram shows the limited similarity of the MF2 forest area with the Dürrenstein Wilderness Area plots, paralleling the significantly reduced degree and diversity of mycophagy at MF2 (Table 4, results of Tukey analysis).

**Table 4:** Differences in number of defecated ECM spore types between the four main small mammal species, forest areas and sessions

Results of MANOVA					
Factor	df	Sum Sq	Mean Sq	*F* value	*P* value
Species	3	33.6	11.2	30.4	<0.001***
Plot	3	6.7	2.2	6.1	<0.001***
Session	1	1.7	1.7	4.6	0.036*
Species/plot	9	6.7	0.7	2.0	0.037*
Species/session	3	3.9	1.3	3.6	0.014*
Plot/session	3	0.3	0.1	0.3	0.838
Species/plot/session	8	2.9	0.4	1.0	0.447
Residuals		450	165.9	0.4	

Results of Tukey multiple comparison of means					
	Mean no. of ECM fungal spore types	Difference	Lower	Upper	*P* value
Species					
*M. glar./A. flav.*	3.3/2.0	0.436	0.263	0.610	<0.001***
*M. glar./S. aran.*	3.3/1.7	0.632	0.860	0.405	<0.001***
*M. glar./S. min.*	3.3/1.5	0.625	0.830	0.419	<0.001***
*A. flav./S. aran.*	2.0/1.7	0.196	0.425	−0.033	0.124
*A. flav./S. min.*	2.0/1.5	0.188	0.396	−0.019	0.089′
*S. aran./S. min.*	1.7/1.5	0.008	0.247	0.262	0.999
Plot					
MF2/DA	1.6/2.1	0.243	0.451	0.035	0.015*
MF1/DA	3.0/2.1	0.084	0.118	0.287	0.705
PF/DA	2.7/2.1	0.035	0.235	0.165	0.969
MF1/MF2	3.0/1.6	0.327	0.122	0.532	<0.001***
PF/MF2	2.7/1.6	0.208	0.006	0.411	0.041*
PF/MF1	2.7/3.0	0.119	0.315	0.077	0.398
Session					
Su/Au	2.2/2.3	0.115	0.225	0.005	0.999
Species: plot (only significant pairs given)					
*M. glar. MF1/M. glar. MF2*	4.4/1.8	0.609	0.211	1.008	<0.001***
*M. glar. PF/M. glar. MF2*	4.0/1.8	0.496	0.078	0.915	0.005**
Species: session (only significant pairs given)					
*M. glar. Su/M. glar. Au*	2.9/3.7	0.311	0.599	0.024	0.023*

Parameters that were significant at *P* = 0.05 are in bold

′* P* = 0.1; * P = 0.05; ** P = 0.01; *** P = 0.001

### Effect of small mammal species, session, year and forest area for single ECM fungal spore types

In *My. glareolus* samples we found all 20 ECM fungal taxa and in samples of *A. flavicollis* we detected 19 of 20 taxa. Samples from the shrew species contained 16 (*S. araneus*) or 17 (*S. minutus*) ECM fungal taxa. In the samples of the rare small mammal species we determined eight (*A. sylvaticus*, *G. glis*, *S. alpinus*) and ten (*Mi. agrestis*) ECM fungal spore types, respectively.

We observed that 11 % of all samples lacked ECM fungal spores, 75 % of all samples contained one to four taxa, and the remaining 14 % contained five to nine taxa of ECM fungi. MANOVA and Tukey post hoc test with following model: no. of ECM spore types ~ Small mammal species × Forest Area × Session (Table 4a) revealed all three variables to have significant influence on the dependent variable. Samples from *My. glareolus* showed the highest number of spore types (*p* < 0.001), but we did not detect significant differences between the other three common small mammal species. For the variable “Plot”, we found samples from the Rosalia Demonstration Forest (MF2) to have significantly lower numbers of spore types, while the three forest areas within the Dürrenstein Wilderness Area did not exhibit significant differences. For the variable “Session” we found no significant differences between summer and autumn samples (Table 4, results of Tukey analysis).

We explored the influence of environmental factors on ECM fungal spore types (Table 1, ESM Tables 1 and 2) by negative binomial regression with the factors “Small mammals species”, “Plot” and “Session” for every spore type separately (Table 5). We restricted this evaluation to data from Dürrenstein Wilderness Area to reduce unaccounted variation resulting from geographical separation and different habitat characteristics. For *Cortinarius* sp. the given set of coefficients was invalid, so we omitted the “Session” factor (in Table 5 denoted as not calculated), to reduce the number of factors.

**Table 5:** Coefficients of negative binomial regression for single ECM types (*z* values calculated with negative binomial regression), data from Dürrenstein Wilderness Area only

	Peziz	Genea	Bals	Truf	Tpub	Easp	Egran	Emur	Espimm	Hneph
Intercept	0.000	−1.306	−2.700**	1.471	−1.848′	−4.192***	0.000	−2.540*	−0.009	−1.447
Species										
* A. flavicollis*	0.000	0.000	0.000	0.000	0.000	0.000	0.000	0.000	0.000	0.000
* My. glareolus*	0.000	4.257***	2.091*	1.551	0.759	3.452***	0.000	4.053***	−1.048	5.845***
* S. araneus*	0.000	−0.467	−1.410	0.000	0.549	−2.952**	0.000	0.000	2.854**	1.900′
* S. minutus*	0.000	−0.154	0.210	0.000	3.502***	−2.941**	0.000	1.406	1.082	−2.770**
Plot										
DA	0.000	0.000	0.000	0.000	0.000	0.000	0.000	0.000	0.000	0.000
MF1	0.000	0.011	−2.123*	2.840**	−1.296	−2.282*	0.000	−2.509*	0.008	0.140
PF	0.000	−0.380	−1.098	1.526	−0.129	−1.982*	0.000	−0.747	0.008	−0.315
Session										
Autumn	0.000	0.000	0.000	0.000	0.000	0.000	0.000	0.000	0.000	0.000
Summer	0.000	−1.299	−1.344	−1.020	2.415*	−0.705	1.262	−0.584	−1.430	1.446
Year										
2006	0.000	0.000	0.000	0.000	0.000	0.000	0.000	0.000	0.000	0.000
2007	0.000	1.304	2.699**	−1.472	1.846′	4.192***	0.000	−1.968′	0.009	1.447

	Russ1	Russ2	Bol	Ccaes	Oast	Mel	Cort	HymCort	Hymhes	Hym1-5
Intercept	6.754***	5.093***	2.178*	1.435	4.316***	4.088***	2.686**	0.001	−2.322*	−3.448***
Species										
* A. flavicollis*	0.000	0.000	0.000	0.000	0.000	0.000	0.000	0.000	0.000	0.000
* My. glareolus*	5.092***	6.359***	4.008***	2.231*	0.250	2.569*	6.234***	0.590	−1.072	3.598***
* S. araneus*	0.557	1.309	0.593	−2.293*	−3.922***	−5.011***	0.414	1.422	−0.754	−3.481***
* S. minutus*	−1.239	1.009	0.930	−4.788***	−4.888***	−5.605***	0.035	0.916	1.350	−4.752***
Plot										
DA	0.000	0.000	0.000	0.000	0.000	0.000	0.000	0.000	0.000	0.000
MF1	0.673	−4.168***	3.138**	−2.653**	1.252	3.479***	1.875′	0.000	1.386	−1.240
PF	3.350***	−3.132**	−1.030	−3.096**	0.314	1.295	0.764	0.000	2.268*	−2.490*
Session										
Autumn	0.000	0.000	0.000	0.000	0.000	0.000	Not calc.	0.000	0.000	0.000
Summer	−0.635	5.311***	0.395	2.293*	5.997***	5.155***		0.000	−0.020	−1.653′
Year										
2006	0.000	0.000	0.000	0.000	0.000	0.000	0.000	0.000	0.000	0.000
2007	−6.755***	5.091***	−2.180*	−1.432	3.065***	−4.004***	−2.687**	−2.175*	−3.641***	3.452***

Parameters significant at *P* = 0.05 are in bold

′* P* = 0.1; * P = 0.05; ** P = 0.01; *** P = 0.001

The factor “Small mammal species” significantly explained variation in observed spore numbers in 15 out of 20 spore types, but each spore type differed in results regarding the single factor levels. According to the *z* values, *My. glareolus* samples contained significantly more spores of 12 spore types than any other of the three common small mammal species (Table 5). Two ECM fungal spore types, *Tuber* aff. *puberulum* and *Russulaceae* 2, were significantly more abundant in samples of *S. minutus* and *S. araneus*, respectively, than in samples from the rodent species, while six types (*E. asperulus*, *Chamonixia caespitosa*, *Melanogaster broomeianus*, *Hysterangium nephriticum*, *Hymenogaster* sp.1–5, *Octaviania asterosperma*) had significantly lower *z* values in *Sorex* samples (Table 5). For the regression factor “Plot” we found that MF and PF had a similar effect on the number of observed fungal spores for ten ECM fungal spore types. In six ECM fungi (*Balsamia* sp., *Elaphomyces asperulus*, *E*. cf. *muricatus*, *Russulaceae* 2, *C. caespitosa*, *Hymenogaster* sp.1–5) calculated *z* values were lower in samples from MF and PF than in samples from DA, while for *T. rufum*, *Russulaceae* 1, *Boletaceae*, *M. broomeianus*, and *H* cf. *hessei* *z* values were higher for samples from MF and PF (Table 5). For most of these spore types, results were significant for both MF and PF, but *Russula* sp.1 and *H.* cf. *hessei* were most abundant in samples from PF and *Hymenogaster* sp.1–5 was least abundant in samples from this plot. *T.* *rufum*, *Boletaceae* and *M. broomeianus* were most abundant in samples from MF, while *Balsamia* sp. and *E*. cf. *muricatus* were least abundant in MF samples. For the remaining ten ECM fungal spore types the factor “Plot” did not explain variation in observed numbers of ECM spores.

The factor “Session” explained spore number variation in five ECM fungi, for all of them we found more spores in Autumn samples. Regarding the sampling years we found significantly more spores of *E.* cf*. muricatus*., *Russulaceae* 1, *Boletaceae*, *M. broomeianus*, *Cortinarius* sp., *Hymenogaster*/*Cortinarius* sp., *H.* cf. *hessei* in samples from 2006, while observed spore numbers of *Balsamia* sp., *T.* aff. *puberulum*, *E. asperulus*, *Russulaceae* 2, *O. asterosperma*, and *Hymenogaster* sp.1–5 were significantly higher in 2007 (Table 5).

### Food web nestedness

**Fig. 4:**
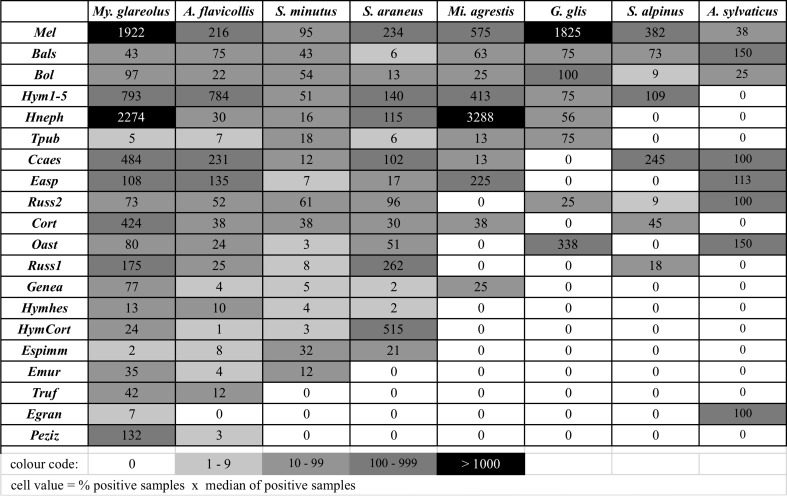
Heat map of ECM fungal spore egestion by small mammal species illustrating the nested arrangement of the mycophagist–ECM fungus network (values = % of positive samples × median of spore counts in positive samples; for abbreviations of fungal taxa see Table 1)

The heat map (Fig. 4) showed an arrangement typically found in nested communities. In our case a subset of fungi was consumed more frequently (indicated by higher values and darker shade) and by more small mammal species, while other ECM fungi seemed to be taken up in low numbers but widespread throughout the small mammal community; and finally there were five ECM fungi (*Pezizales* sp., *Tuber rufum*, *Elaphomyces granulatus*, *Hymenogaster*/*Cortinarius* sp., *Hymenogaster* cf. *hessei*) which were restricted to only a few small mammal species.

The calculated system temperature was *T* = 3.76. The calculated mean temperatures of three null models were *T*_1_ = 32.60, *T*_2_ = 30.92 and *T*_3_ = 22.32. The statistic comparison resulted in rejection (*p* < 0.001 for all three null models) of the null hypothesis (*H*_0_ = matrix of food web randomly created) and supported hypothesis 4.

## Discussion

### Varying degree of mycophagy in the small mammal community

The presence of fungal spores of ECM fungi in samples of all eight small mammal species studied confirms and extends earlier reports on mycophagy in the vole *My. glareolus*, the murid *A. flavicollis* (Blaschke and Bäumler 1989) and, as shown very recently, in the insectivores *S. araneus* and *S. minutus* (Kataržytė and Kutorga 2011). The data about mycophagy in *G. glis* and *S. alpinus* are the first of their kind, to the best of our knowledge.

Significant differences in numbers of fungal spores ingested by different small mammal species indicate a species-specific degree of mycophagy in the small mammal community of Central European mountain forests, confirming hypothesis 1. Furthermore, mycophagy of at least four of the investigated small mammal species varies seasonally and/or between forest areas, so hypotheses 2 and 3 are supported as well.

Our observations on the distribution of spores of hypogeous fungi in the faeces of all eight small mammal species parallel results from other continents. There is one species (*My. glareolus*) consuming a considerable variety and amount of ectomycorrhizal fungi across all forest types and trapping sessions and there are many others which use this food source periodically or with a narrower range of consumed species (Fig. 4). Vernes and Dunn (2009) found the same pattern across a landscape gradient in eastern Australia, where the bush rat (*Rattus fuscipes*) as the main mycophagous species was accompanied by many other species with a lesser degree of mycophagy.

*My. glareolus* exhibits the highest degree of mycophagy in terms of quantity and species richness, but its feeding habits appear to be less specialised on fungi compared to its North-American cousins *My. californicus* and *My. gapperi*, which are considered obligate mycophagists (Ure and Maser 1982). The number of spores observed in the *My. glareolus* samples varies between sessions, forest types and individual samples, and there are other food items present in varying amounts throughout the year. The important seasonal variation in the quantity and diversity of ECM fungal species consumed by *My. glareolus* suggests that supply of this food source is too unpredictable in the habitats investigated to allow high specialisation. However, given the overall abundance and diversity of fungal spores ingested, *My. glareolus* might be regarded as preferentially mycophagous (Claridge and Trappe 2005).

For *A. flavicollis* fungi seem to be an important food source in times of abundance, but with only one tenth of ECM spores observed compared to *My. glareolus* the species has to be regarded as casually or opportunistically mycophagous (Maser et al. 2008). Given the less burrowing lifestyle of *A. flavicollis* this is not surprising; however, some animals defecated substantial amounts of fungal material (up to 5,000 spores per 50 fov). We can therefore conclude that fungi are consumed in considerable quantities, when available, but *A. flavicollis* does not rely on them as a food source.

We found significantly less ECM spores in the faeces of all three shrew species than in samples from the two main rodent species, but there are some samples with very high numbers of ECM fungal spores. While the number and aggregation of ECM spores in the rodent samples indicate mycophagy, it remains unclear whether the shrews forage actively for fungi. Their insectivorous feeding habit might cause them to ingest fungal spores when they prey on mycophagous invertebrates (Fogel and Trappe 1978). Indirect spore uptake has been shown for other insectivorous species, e.g. *Antechinus* spp. (Vernes 2007) and is considered as accidental mycophagy (Claridge and Trappe 2005). But, the high spore numbers we observed in some samples rather indicate direct consumption of fungal fruit bodies (especially *Tuber* aff. *puberulum*, *Russulaceae* 2), albeit possibly infested with insect larvae. Therefore *Sorex* spp. might better be considered selective occasional mycophagists. Efficiency of spore dispersal can be assumed to depend on a variety of behavioural properties, such as microhabitat preferences and home range size. Even as secondary consumers, shrews can act as spore vectors by distributing the viable spores over a larger distance than the primary invertebrate consumers might do. Thus, shrews might play an underestimated role in spreading fungal spores.

*A. sylvaticus*, *G. glis* and *Mi. agrestis* can be regarded as opportunistically mycophagous. Spore numbers found in the scats of *A. sylvaticus* are within the same range as found for *A. flavicollis*. With regard to mycophagy, no niche differentiation could be detected between the two *Apodemus* species. *G. glis* seems to descend to the forest floor to dig for truffles despite its mainly arboreal lifestyle, like the opportunistically mycophagous brushtail possums (*Trichosurus vulpecula*) of Southeastern Australia (Claridge and Lindenmayer 1998) or various squirrel species (Bertolino et al. 2004; Vernes et al. 2004). Owing to its rather large home range (up to 7 ha for males; Ściński and Borowski 2008), *G. glis* might be an important long distance vector.

Regarding the ecological niche of *Mi. agrestis* and *P. subterraneus* (strictly ground-dwelling vole species with a lifestyle comparable to that of *My. glareolus*; Mitchell-Jones et al. 1999), we expected higher amounts of ECM spores in their samples. From the overall results it is clear that their low degree of mycophagy compared to *My. glareolus* can not be explained by a shortage of ECM fungi during the sampling sessions, but more samples are needed to draw conclusions. We suggest sampling in areas of known abundance of these species to achieve representative sample sizes.

### Diversity of ECM fungi consumed

The majority of samples contained between one and four ECM fungal spore types, indicating that small mammals consume a variety of ECM fungi if available. This polyphagous feeding habit reduces dependence on differing fruiting times of the ECM species, making the fungal food source more reliable. Other potential advantages might be a more balanced nutrition as well as reduced searching effort.

Only two of the hypogeous fungal taxa detected during this study (*Hysterangium* *nephriticum* and *Elaphomyces muricatus*) had been discovered during previous surveys (Kovacs 2001) in the Dürrenstein Wilderness Area. The study by Kovacs (2001) revealed a high diversity of epigeous ECM fungi in the primeval forest area, but the fact that we found a larger proportion of spores of hypogeous fungi shows that small mammals prefer those, even if mushrooms are abundant. The reason for this might be the more stable supply of truffles or the reduction of toxicity in hypogeous fungi (Claridge and Trappe 2005).

The relatively low frequency of spores of hypogeous ascomycetes of the genus *Elaphomyces* in the faecal samples is in contrast to fortuitous ascocarp records, but can be explained by the specific dispersal strategy of this genus: unlike other hypogeous fungi, the spores form a powdery mass, which is exposed to the wind during manipulation by mycophagists that typically feed on the peridium (Maser et al. 2008).

### Forest succession and diversity of small mammal and hypogeous fungal communities

The four investigated forest types differ regarding both the small mammal and the fungal components of the mycophagy network. The observed variations in abundance, species richness and composition of egested spores of hypogeous fungi indicate site-specific ECM communities to which the mycophagists can adapt.

The feeding habit of *My. glareolus*, the key mycophagist, does not differ significantly between the primeval forest and the sustainably managed forest plots in the Dürrenstein Wilderness Area, indicating that forest management practices can be compatible with the persistence of mycophagous relationships. On the succession plot (DA) mycophagy by *My. glareolus* is slightly lower in terms of spore abundance and diversity, but these differences are statistically not significant. The structurally diverse succession plots, with a tessellate pattern of graminoid and herbal vegetation and patches of forest regeneration, is likely to provide a diversity of alternative food sources, potentially reducing the fidelity of the mycophagists to their fungal food source, albeit without challenging the persistence of the mycophagy network. Six spore types of hypogeous fungi were observed to be most abundant in samples from the DA, while several others were found to be less abundant than in more mature forests (Table 5). The differences in community composition between the DA and the MF1 and PF (Fig. 3) suggest that a specific community of hypogeous fungi is associated with the successional stage of the DA.

Reduced mycophagy in the Rosalia Demonstration Forest plots (MF2) may in part be due to the composition of the dense ground vegetation of mostly arbuscular mycorrhizal host plants (grasses, ferns, bramble). An abundant ground vegetation appears to provide alternative food sources for *My. glareolus*, sustaining high population densities but reducing its dependency on fungi, and can compete with trees and their ectomycorrhizal associates for water and nutrients (e.g. Dodet et al. 2011) potentially reducing the resources allocated to sporocarp production. This leads us to the hypothesis that more or less mycophagous feeding habits may be part of feedback loops which result in either rapid regeneration of predominantly ECM forest or long-term persistence of grassy and shrubby vegetation.

Several additional factors may account for differences in intensity of mycophagy and in diversity of hypogeous fungi consumed that we observed between the two study regions. The managed forest plots in both sites are comparable with regard to tree species composition, tree age and practices of forest management, but in the Rosalia Demonstration Forest they are surrounded by intensively managed forests in a densely populated area. The fungal species richness of the investigated managed forest in the Dürrenstein Wilderness Area (MF1) shows the positive impact of large extensions of sustainably managed forests and the close proximity of primeval forest, where mycophagists potentially vector ECM fungal spores between managed and unmanaged forest. Furthermore, the humid, suboceanic climate is likely to promote ECM fungal diversity and continuous productivity in the Dürrenstein Wilderness Area, while fungal sporocarp production is limited by periods of draught characteristic of the more continental climate of the Rosalia Demonstration Forest.

### Mutualistic network structure

The limitations of the taxonomic resolution achievable by light microscopy and uncertainties connected to rarer spore types may result in an oversimplified representation of the actual fungi–mycophagist network, but general patterns are clearly visible. The high degree of nestedness suggests that the trophic relationships involving generalists and specialists are arranged in a non-random way i.e. that they are highly organised. This result indicates that the analogies of mycophagy with other resource–service mutualistic networks such as pollination or frugivory result in very similar structural characteristics, and that conclusions reached for more easily accessible networks (e.g. plant–pollinator or plant–frugivore; Bascompte et al. 2003) might also apply to the fungi–mycophagist relationship. Nestedness can provide feedback loops and promote diversity by increasing the number of coexisting species (Bastolla et al. 2009). Mycophagy is considered to enhance functionality and resilience of forests (Johnson 1996; Claridge 2002), and nestedness appears to contribute to these ecosystem functions.

## Conclusions and management considerations

The animal and fungal species involved in mycophagy networks differ among continents, but some fundamental characteristics appear to be surprisingly similar. Phylogenetically diverse communities of animal species (Johnson 1996) disperse a highly diverse assemblage of fungal species, among which the typically ECM hypogeous fungi are predominant. Varying levels of specialisation and dependency are found on the higher trophic level, while hypogeous fungi fully rely on animal vectors for dispersal.

In the investigated Central European forest ecosystems no obligate mycophagist is present, but all small mammal species studied are mycophagous to some extent. The most active mycophagists are not endangered, unlike Australian mycophagous species (e.g. Johnson 1996; Green et al. 1999; Vernes 2003). The dietary ecology of small mammals including *Sorex* spp. and *G. glis* appears to be more versatile than previously reported. The exclusive view of rodents as predators of tree seeds and seedlings needs to be revised.

Hypogeous ECM fungi seem to be present in considerable diversity and abundance. At least 14 certainly hypogeous fungal taxa depend on dispersal by mycophagists in the investigated areas, 12 of them reported for the first time. Furthermore, the unexpected high frequency of *Chamonixia caespitosa* spores shows that mycophagy studies can provide new data about the ecology and distribution of hypogeous fungi, one of the least known groups of macrofungi. More data about these partly red-listed species are needed not least for a better assessment of their conservation status.

In contrast to mycorrhizal symbiosis, mycophagy is not yet widely acknowledged as a process contributing to forest vitality, productivity and resilience. This investigation demonstrates that a non-negligible part of the ectomycorrhizal communities of managed and unmanaged forests in different successional stages relies on animal dispersal. A specific community of ectomycorrhizal hypogeous fungi appears to be associated with younger trees in forest regeneration sites after large-scale disturbance. We hypothesise that (1) the availability of animal vectored ectomycorrhizal inoculum is important for forest regeneration particularly in situations approximating primary succession and (2) that mycophagy interacts with successional trajectories leading either to rapid development of predominantly ECM forest or long-term persistence of predominantly arbuscular mycorrhizal ground vegetation.

Impact of timber harvest on fungi and mammal diets was studied extensively in the Northwestern United States (Carey and Harrington 2001; Carey et al. 2002; Luoma et al. 2004; Gitzen et al. 2007). As we found similar relationships between small mammals and fungi in Central Europe, we can adopt the recommendations inferred. A sustainable management retaining mature trees and protecting the forest soil limits disturbance to a level that does not impair the persistence of mycophagy networks, leaving intact sources of mycorrhizal fungi and alternative food for small forest mammals (Dell 2002; Wiensczyk et al. 2002). Additionally, Wiensczyk et al. (2002) strongly recommend the retention of coarse woody debris, because Amaranthus et al. (1994) found that fruiting of hypogeous fungi was linked to the presence of coarse woody debris. Maintaining a natural composition of both small mammal and hypogeous fungal communities will promote forest health and contribute to forest resilience (Jacobs and Luoma 2008).

## Electronic supplementary material

Below is the link to the electronic supplementary material.

Supplementary material 1 (DOC 711 kb)

Supplementary material 2 (DOC 1366 kb)
