# Supplementary material for: The interrelationship of mycophagous small mammals and ectomycorrhizal fungi in primeval, disturbed and managed Central European mountainous forests
Source: Oecologia. 2012 Apr 1;170(2):395–409. doi: 10.1007/s00442-012-2303-2 (PMC3439606; doi:10.1007/s00442-012-2303-2)
Supplement: Supplementary file 1 — Supplementary material 1 (DOC 711 kb) [file 442_2012_2303_MOESM1_ESM.doc]

**Electronic Supplementary Material (ESM) 1**

**The interrelationship of mycophagous small mammals and ectomycorrhizal fungi in primeval, disturbed and managed Central European mountainous forests**

Susanne Schickmann, Alexander Urban, Katharina Kräutler, Ursula Nopp-Mayr, Klaus Hackländer

**ESM1:** Numbers of spores of different taxa of ECM fungi egested by small mammal species. min/max = minimum/maximum no of spores per sample; %ps = percent of positive samples; mps = median spore number in positive samples. For abbreviations of fungal taxa see tab. 1.
