# Supplementary material for: The interrelationship of mycophagous small mammals and ectomycorrhizal fungi in primeval, disturbed and managed Central European mountainous forests
Source: Oecologia. 2012 Apr 1;170(2):395–409. doi: 10.1007/s00442-012-2303-2 (PMC3439606; doi:10.1007/s00442-012-2303-2)
Supplement: Supplementary file 2 — Supplementary material 2 (DOC 1366 kb) [file 442_2012_2303_MOESM2_ESM.doc]

**Electronic Supplementary Material (ESM) 2**

**The interrelationship of mycophagous small mammals and ectomycorrhizal fungi in primeval, disturbed and managed Central European mountainous forests**

Susanne Schickmann, Alexander Urban, Katharina Kräutler, Ursula Nopp-Mayr, Klaus Hackländer

**ESM2:** Numbers of spores of different taxa of ECM fungi egested by the most frequent small mammal species for each of the four investigated forest types (abbreviations as in ESM1).
